# Gomberg’s Earlier “Instance of Trivalent Carbon”

**DOI:** 10.1021/jacs.5c21781

**Published:** 2026-02-06

**Authors:** Christopher Grainger, St. John Whittaker, Dencie Desrosiers, Stephanie S. Lee, Alexander G. Shtukenberg, Bart Kahr

**Affiliations:** † Department of Chemistry and Molecular Design Institute, 5894 New York University, 24 Waverly Place, New York, New York 10003, United States

## Abstract

In this journal, Moses Gomberg’s 1900 revelation, “An Instance of Trivalent Carbon: Triphenylmethyl”, lauded on a centennial National Historic Chemical Landmark for challenging the “prevailing belief that carbon can only have four bonds”, shifts its place in our imaginations as the facts given here are accommodated. In 1898 Gomberg presumed that he had made a molecular complex of bromotriphenylmethane and two neutral I_2_ molecules. But he was mistaken. Instead, Gomberg produced a mixture of three persistent single crystals of the triphenylmethyl cation before he published his aforementioned, controversial paper. Trigonal carbon coordination was the crack in the valency rules that had organized chemistry prior to the invention of quantum mechanics. Gomberg did not recognize the wealth of trivalent carbon compounds he had in hand before the proposition of the radical and corresponding cation thereafter. This work alludes to a counterfactual history.

## Graphical abstract

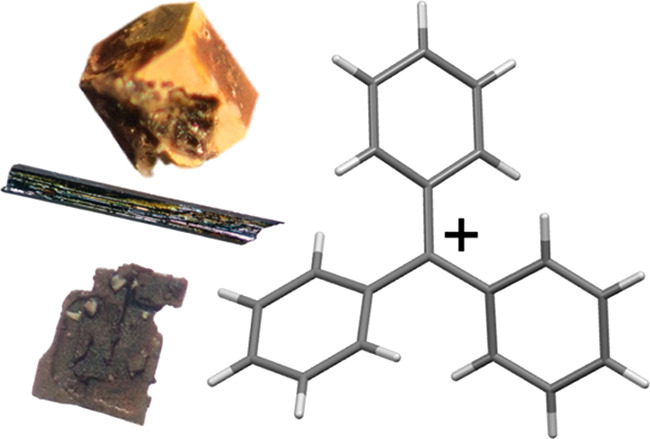

Few papers in the history of organic chemistry^1^ were as impactful as Gomberg’s “An instance of trivalent carbon: Triphenylmethyl”.^2,3^ In proposing a stable free radical, Gomberg struck down valency rules that governed structural chemistry prior to an electronic theory of bonding. McBride told the story of the turmoil created by Gomberg’s proposal.^4^ This history has been updated by Eberson, with records now available from the Royal Swedish Academy of Science Archive.^5^

McBride stressed that the “monumental development of structural organic chemistry based on the hypothesis of tetravalent carbon” was shown by Gomberg’s landmark achievement to be incomplete, while at the same time the proposal of free radicals aroused great skepticism.^6^ Yet, the triphenylmethyl (TPM) radical was later cemented as the TPM cation came into focus.^7^ The product of chlorotriphenylmethane and sulfuric acid was salt-like,^8^ with the properties of an electrolyte.^9,10^ Gomberg and Cone^11^ isolated the perchlorate salt of the TPM carbocation which could be recrystallized as spectacular octahedra. The TPM^+^·ClO_4_^–^ crystal structure was worked out in 1965 ^12^ Hofmann and Kirmreuther’s crystallization conditions.^13^ A definitive history of the triphenylmethyl cation was given by Nenitzescu.^14,15^

In 1898, Gomberg reported “A periodide of triphenylbromomethane”^16^ by treating the title compound with a benzene solution of iodine. Blue-green iridescent crystals resembled to Gomberg the periodide of quinine discovered by Herapath^17^—known as herapathite^18–20^ —which Land later utilized to construct the first synthetic linear polarizers,^21^ launching the Polaroid Corporation. Gomberg gave the composition of his product as (C_6_H_5_)_3_CBr·2I_2_, a molecular complex. We set out to prepare Gomberg’s iridescent crystals given the possibility of another crystal, simpler than herapathite, that could have likewise been fashioned into polarizers before the invention of Land.^22^ Ours was a historical reinvestigation requiring contemporary experiments. But what we found was more surprising to us than another periodide-based linear polarizer. We characterized a group of stable crystalline triphenylmethyl cations with planar trigonal carbon atoms that Gomberg had grown likewise prior to 1900. Bromotriphenylmethane (0.511 g, 1.58 mmol) and iodine (1.055 g, 4.16 mmol) were each dissolved in 20 mL of benzene and the solutions were combined according to Gomberg.^16^ A “dark granular precipitate” was “thrown down” from the resultant deep purple solution. The solids were washed with benzene and vacuum-dried.

Single crystal X-ray diffraction data was obtained using a Bruker D8 SMART APEX II diffractometer equipped with a PHOTON–II-C14 detector. The X-ray beam (Mo Kα, λ = 0.71073 Å) generated from an INCOATEC microfocused source was monochromated. The crystal was cooled to 100(2) K with an Oxford Cryosystems 700+ Cooler. The crystals were mounted on 0.2 mm MiTeGen MicroMount loops with Type B immersion oil (Cargille Laboratories). The data sets were collected with omega and phi scans and processed with the INTEGRATE program of the APEX4 software for reduction and cell refinement.^23^ Multiscan absorption corrections were applied by the SCALE program for the area detector. Three structures were solved by intrinsic phasing methods (SHELXT) and the structure models were completed and refined using the full-matrix least-squares methods on *F* ^2^ (SHELXL).^24^ Non-hydrogen atoms were refined with anisotropic displacement parameters, and hydrogen atoms were placed in idealized positions (C–H = 0.95–1.00 Å) and included with *U*_iso_(H) = 1.2.

Dark needles with structure **1** (C_19_H_15_Br_2_I_3_, Figure 1A) were characterized in the space group *Pnna* (#52), lattice constants *a* = 17.9244(7) Å, *b* = 12.6300(5) Å, *c* = 9.4066(3) Å, *V* = 2129.5(1) Å^3^, *R*_1_ = 2.12% (*I* > 2*s*_*I*_), *wR*_2_ = 4.92% (all data), CCDC number 2431723 (further details in Supporting Information (Table S1)). However, the crystals could not be described as a molecular complex of iodine and bromotriphenylmethane as Gomberg had presumed. Rather, TPM cations sit on dyad axes; their symmetry independent C–C_methyl_–C angles (Table S2) are consistent with trigonal planar coordination. The dihedral angles between the plane of coordination of the central C and the phenyl rings were 45.7(2)°, 27.4(2)°, and 27.4(2)°, consistent with other TPM cations.^25,26^ The negative charge resides in BrIBr^–^ anions that exhibit a typical near-linear (176.89(2)°) geometry.^27^ The BrIBr^–^ ions are loosely bridged by I_2_ molecules, a motif that has been recorded previously.^28–30^ Many other polyhalogen networks of differing composition are recorded in literature.^27,31^ The I–Br bond length of 2.7083(3) Å is typical, as are ion–molecule (BrIBr^–^–I_2_) distances between nearest atoms of 3.2483(4) and 3.8354(4) Å.

**Figure 1:**
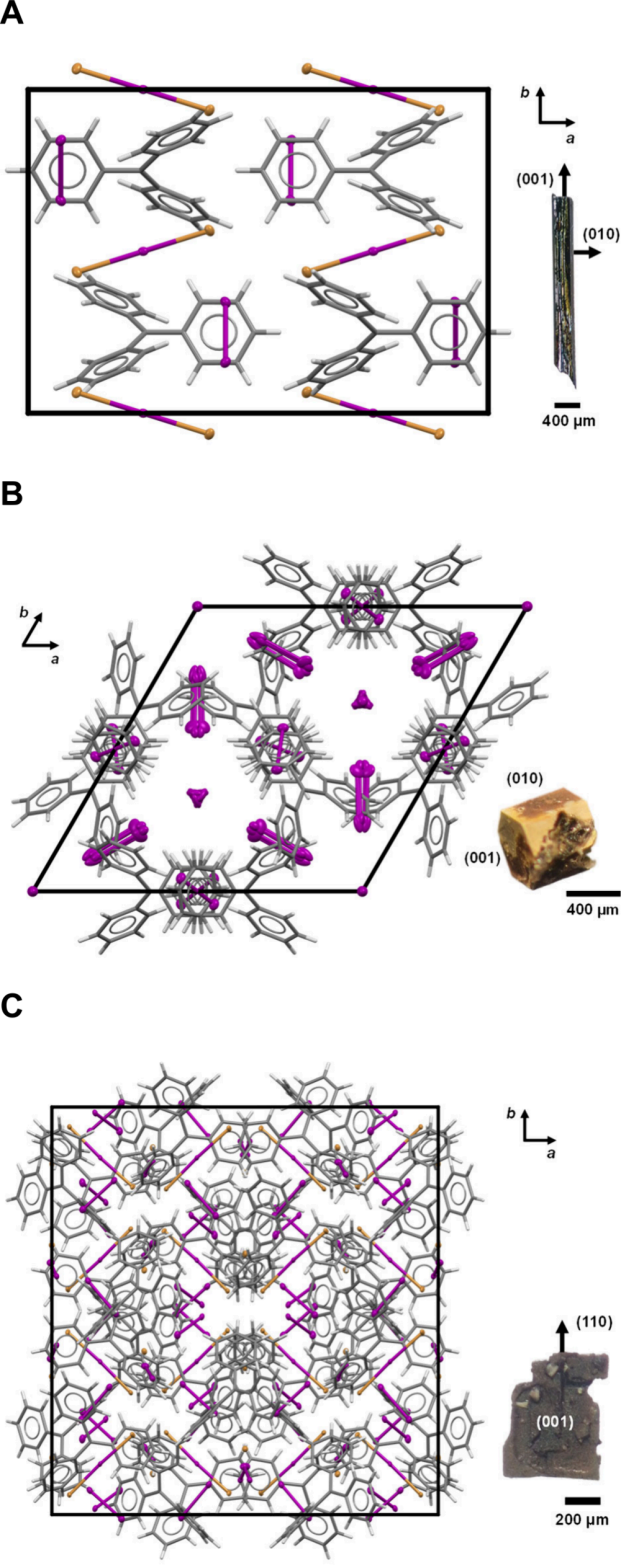
Crystal structures **1** (A), **2** (B), and **3** (C) each viewed along *c*. TPM cations are stylized as capped sticks and halogen atoms as ellipsoids. Indexed images of crystals at right of structures.

We were nevertheless perplexed by the composition of our crystal. Structure **1** had ratios of I:Br:TPM = 3:2:1, distinct from the composition given by Gomberg, 4:1:1. The ratio of bromine to iodine in our structure was too large, and the ratio of TPM to iodine was too small. Any student of Gomberg would be suspicious of this discrepancy.

Gomberg was a skilled analyst, as McBride emphasized.^4^ He had analyzed the iodine in his crystals in several different ways, in most cases titrating ethanolic solutions of his crystals against sodium thiosulfate.^16^ He then determined the bromine stoichiometry by reaction with silver nitrate. We were thus confident that there must be other crystals of differing composition lurking in Gomberg’s dark precipitate. We identified crystals with stout hexagonal prismatic morphologies (Figure 1B) and others as irregular plates (Figure 1C) grown alongside the acicular crystals of structure **1** (Figure 1A). Gomberg had presumed that these were the same solids.

In search of more iodine, we solved structure **2** (Figure 1B) of the hexagonal prisms: space group *P*3̅*c*1 (#165), *a* = 16.4645(4) Å, *c* = 19.1960(7) Å, *V* = 4506.5(3) Å^3^, *R*_1_ = 3.65% (*I* > 2*s*_*I*_), *wR*_2_ = 8.66% (all data), CCDC number 2431721. Structure **2** is another salt of the TPM cation: a propeller that again sits on a dyad axis. The symmetry-independent C–C_methyl_–C angles are consistent with the trigonal planar coordination. The dihedral angles between the phenyl rings and the central coordinating plane (Table S2) are comparable to Gomberg’s structure **1**. The formula for this complex, C_17.72_H_14.72_Br_0.14_I_5.45_, was indeed rich in iodine but is nonstoichiometric due to several kinds of disorder.

Structure **2** has two unique semi-infinite halogen chains. One lies along (0, 0, *z*) featuring disordered linear arrays of triiodide anions, essential for charge balance. The second halogen chain positioned at (1/3, 2/3, *z*) consists of alternating X^–^ and I_2_; the X^–^ site was refined as Br:I = 0.29(1):0.71(1). The I_2_ molecules display a misalignment around the 3-fold axis at one end.

Two unique connected branches of disordered iodine molecules run tangentially to the chain at the anion site. One site interchanges neutral benzene with iodine in a ratio of C_6_H_6_:I_2_ = 0.771(2):0.229(2). The second features a positional disorder of two I_2_ molecules in a ratio of 0.64(2):0.36(2). Coppens^32^ proposed a limit of 3.30 Å for iodine covalent interactions. More recently, “secondary bonding” for I–I distances in the range 3.4–3.7 Å has been investigated quantum chemically,^33^ though this idea was first introduced in a broader context.^34,35^ The longer regime describes interactions at commonly seen interatomic distances that may be partly covalent. Many such polyiodide systems exhibit these characteristic secondary bonding interactions,^36^ leading to anions as large as I_29_^3–^.^37^ Evidence for covalency in polyiodides up to 3.5 Å has been articulated.^38^ Structure **2** exhibits a wide range of bond distances between the parallel linear chains and both disordered I_2_ molecules. Many of these distances correlate with typical secondary bonds; in total, they form a 3D halogen network (Figure S3).

A second hexagonal prism of structure **2**, prepared in the same manner as the first, gave slightly different lattice constants: *a* = 16.4316(5) Å, *c* = 19.1660(9) Å, *V* = 4481.5(3) Å^3^, *R*_1_ = 4.33% (*I* > 2*s*_*I*_), *wR*_2_ = 11.38% (all data), CCDC number 2431722, and a slightly different formula (C_17.58_H_14.58_Br_0.23_I_5.41_). This second crystal of structure **2** had more bromine, I:Br ∼ 24:1, as opposed to 39:1 in the original crystal. Thus, structure **2** varies in composition between individual crystals but still contains a greater percentage of iodine than Gomberg measured.

Next, we characterized the misshapen plates, structure **3** (Figure 1C), which turned out to be another bromoiodide of a TPM cation. Solving the crystal structure of this compound was a challenge due to unresolved twinning. The best model corresponds to a space group *C*2/*c* (#15) with lattice constants: *a* = 25.553(2) Å, *b* = 25.635(2) Å, *c* = 29.682(2) Å, *V* = 19347(2) Å^3^, *R*_1_ = 8.73% (*I* > 2*s*_*I*_), *wR*_2_ = 22.39% (all data). The residual electron density map shows eight strong (8–12 electron) peaks that can be viewed as four I_2_ molecules superimposed on one aryl ring of four symmetry independent TPM molecules in the asymmetric unit (Figure S4, Table S4). These maxima cannot be accommodated within the current structural model. The formula (C_38_H_30_Br_3_I_9_) presents an integer I:Br:TPM ratio of 9:3:2. Four unique TPM geometries are present in this structure, although they are comparable and typical for trigonal cations. BrIBr^–^ anions balance some of the cation charge, with expected near-linear geometries (176.62(4)° and 176.82(4)°). Br^–^ anions coordinated with six I_2_ molecules (anion–molecule distances 3.138(3) < *d* < 3.359(3) Å) counter the remaining cationic charge. The excess I_2_ serves to increase the relative proportion of iodine compared to structure **1**. Raman spectra at low frequency reflecting the halogen–halogen bonding are given in Figures S5–S7.

Accounting for nonequimolar ratios of the three triphenylmethyl cation crystals that Gomberg prepared, his formula (C_6_H_5_)_3_CBr·2I_2_ would have been sensible. Gomberg had measured a TPM:I ratio of 4.07 by thiosulfate titration.^16^ We repeated his analysis. Approximately 100 mg of dried crystals were weighed and dissolved in acetone before the addition of 50 mL of deionized water, potassium iodide (0.5 g, 3 mmol), and 2–3 drops of aqueous starch. These were titrated against a 0.04 M aqueous sodium thiosulfate solution. Our titration of the crude precipitate gave an equivalent ratio of 3.99. Supporting powder X-ray diffraction data was collected for the mixtures of crystals and analyzed by Pawley and Rietveld refinements (Figures S8-9).

The mystery revealed itself; Gomberg had in fact unknowingly been in possession of three crystalline triphenylmethyl compounds, years before his “instance of trivalent carbon”. One could ask whether Gomberg’s struggles to convince his colleagues of the veracity of hypovalent carbon would have been easier had he reasoned from isolable carbocations rather than from the fleeting intermediate in an equilibrium with its dimer, itself a controversial structure.^4^ Of course, we can ask but not answer. A revisionist history of science can be imagined with new evidence in hand, but counterfactual histories are not real; they can only alert us to alternatives that may pass before our eyes, unnoticed.

## Supplementary Material



## Notes

Tabulated refinement details, the refined geometries of all TPM cations, a figure displaying disorder in the iodine network of structure **2**, as well as details of an alternative refinement for structure **3**; Raman spectroscopy data corresponding to each structure; powder X-ray diffraction patterns and fitted curves (PDF)

## References

[1] American Chemical Society , “Moses Gomberg and the Discovery of Free Radicals,” National Historic Chemical Landmark, Dedicated June 25, 2000, at the University of Michigan in Ann Arbor, Michigan.

[2] Gomberg M. (1900). An Instance
of Trivalent Carbon: Triphenylmethyl. J. Am.
Chem. Soc..

[3] Gomberg M. (1900). Triphenylmethyl,
ein Fall von dreiwerthigem Kohlenstoff. Ber.
dtsch. chem. Ges..

[4] McBride J. M. (1974). The Hexaphenylethane
Riddle. Tetrahedron.

[5] Eberson L. Moses Gomberg and the Nobel Prize. In Culture of Chemistry; Hargittai, B., Hargittai I., Eds.; Springer US: Boston, MA, 2015; pp 283–289, DOI: 10.1007/978-1-4899-7565-2_53.

[6] Schoepfle C. S., Bachmann W. E. (1947). Moses Gomberg 1866–1947. J. Am. Chem. Soc..

[7] Norris J. F. (1901). On the
Nonexistence of Trivalent Carbon. Am. Chem.
J..

[8] Kehrmann F., Wentzel F. (1901). Ueber die basischen
Eigenschaften des Kohlenstoffs
und die Constitution des sogenannten Triphenylmethyls. Ber. dtsch. chem. Ges..

[9] Walden P. (1902). Ueber die
basischen Eigenschaften des Kohlenstoffs. Ber.
dtsch. chem. Ges..

[10] Baeyer A., Villiger V. (1902). Dibenzalaceton und
Triphenylmethan Ein Beitrag zur
Farbtheorie. Ber. dtsch. chem. Ges..

[11] Gomberg M., Cone L. H. (1904). Ueber Triphenylmethyl. Ber. dtsch.
chem. Ges..

[12] Gomes
De Mesquita A. H., MacGillavry C. H., Eriks K. (1965). The Structure of Triphenylmethyl
Perchlorate at 85°C. Acta Crystallogr..

[13] Hofmann K. A., Kirmreuther H. (1909). Carboniumperchlorate. Ber. dtsch. chem. Ges..

[14] Nenitzescu, C. D. Carbonium Ions; John Wiley & Sons, New York, 1968.

[15] Streith J. (2018). C. R. Chimie.

[16] Gomberg M. (1898). A Periodide
of Triphenylbrommethane. J. Am. Chem. Soc..

[17] Herapath W. B. (1852). On the
Optical Properties of a Newly-discovered Salt of Quinine. Philos. Mag. S..

[18] Kahr B., Freudenthal J., Phillips S., Kaminsky W. (2009). Herapathite. Science.

[19] Knowles K. M. (2009). Herapathite
– The First Man-Made Polarizer. Philos.
Mag. Lett..

[20] Liang L., Rulis P., Kahr B., Ching W. Y. (2009). Theoretical
Study
of the Large Linear Dichroism of Herapathite. Phys. Rev. B.

[21] Land E. H. (1951). Some Aspects
of the Development of Sheet Polarizers. J. Opt.
Soc. Am..

[22] Kahr, B. ; Knowles, K. M. Polarizing Films. In Iodine Chemistry and Applications; Kaiho, T. , Ed.; Wiley, 2014; pp 479–488, DOI: 10.1002/9781118909911.ch26.

[23] APEX 4; Bruker AXS Inc.: Madison, WI, 2020.

[24] Sheldrick G. M. (2015). SHELXT
– Integrated Space-group and Crystal-structure Determination. Acta Cryst. A. Found. Adv..

[25] Garratt S., Guerrero A., Hughes D. L., Bochmann M. (2004). Arylzinc Complexes
as New Initiator Systems for the Production of Isobutene Copolymers
with High Isoprene Content. Angew. Chem., Int.
Ed..

[26] Smith J. C., Ma K., Piers W. E., Parvez M., McDonald R. (2010). A New Weakly Coordinating
Aluminate Anion Incorporating a Chelating Perfluoro-bis-anilido Ligand. Dalton Trans..

[27] Sonnenberg K., Mann L., Redeker F. A., Schmidt B., Riedel S. (2020). Polyhalogen
and Polyinterhalogen Anions from Fluorine to Iodine. Angew. Chem., Int. Ed..

[28] Abdelbassit M. S., Curnow O. J., Dixon M. K., Waterland M. R. (2019). Rational
Synthesis, Structures and Properties of the Ionic Liquid Binary Iodine-Bromine
Octahalide Series [I_n_Br_8-n_]^2–^ (n = 0, 2, 3, 4). Eur. J. Chem..

[29] Parlow A., Hartl H. (1985). Synthese und Strukturuntersuchung von Polyhalogeniden im System Iod/Brom/Syntheses
and Structure Analyses of Polyhalides in the System Iodine/Bromine.
Z. Naturforsch. B: Chem. Sci..

[30] Janczak J., Kubiak R. (2003). Sandwich-type Niobium­(V)
Diphthalocyaninato Complexes
‘Stapled’ by Two Inter-Ligand C-C σ-bonds. Synthesis
and Structural Investigations of Two New Phthalocyaninato Complexes:
[NbPc_2_]­(IBr_2_) and [NbPc_2_]­(IBr_2_)·I_2_. Polyhedron.

[31] Babu R., Bhargavi G., Rajasekharan M. V. (2015). Polyhalide Ions (Br_8_
^2–^ and I_2_Br_6_
^2–^) and Chains (···Br^
_3_–^···Br_2_··· and ···IBr^
_4_–^···) Stabilised by Using
Cu­(dafone)_3_
^2+^ as Counter Ion (dafone = 4,5-Diazafluoren-9-one). Eur. J. Inorg. Chem..

[32] Coppens, P. Structural Aspects of Iodine-Containing Low-Dimensional Materials. In Extended Linear Chain Compounds; Miller, J. S. , Ed.; Springer US: Boston, MA, 1982; pp 333–356, DOI: 10.1007/978-1-4613-3249-7_7.

[33] Kloo L., Rosdahl J., Svensson P. H. (2002). On the Intra- and Intermolecular
Bonding in Polyiodides. Eur. J. Inorg. Chem..

[34] Bent H. A. (1968). Structural
Chemistry of Donor-Acceptor Interactions. Chem.
Rev..

[35] Alcock N. W. (1972). Secondary
Bonding to Nonmetallic Elements. Adv. Inorg.
Chem. Radiochem..

[36] Svensson P. H., Kloo L. (2003). Synthesis, Structure, and Bonding
in Polyiodide and Metal Iodide-Iodine
Systems. Chem. Rev..

[37] Tebbe K. F., Buchem R. (1997). The Most Iodine-Rich
Polyiodide Yet: F_3_I_29_
*Angew*. Chem. Int.
Ed..

[38] Savastano M., Bazzicalupi C., Bianchi A. (2022). Novel Cyclen-polyiodide Complexes:
A Reappraisal of I-I Covalent and Secondary Bond Limits. Dalton Trans..

