# Supplementary material for: Gomberg’s Earlier “Instance of Trivalent Carbon”
Source: J Am Chem Soc. 2026 Feb 6;148(6):5905–8. doi: 10.1021/jacs.5c21781 (PMC12921861; doi:10.1021/jacs.5c21781)
Supplement: Supplementary file 1 [file ja5c21781_si_001.pdf]

Gomberg's Earlier "Instance of Trivalent Carbon" Supporting Information

Christopher Grainger, St. John Whittaker, Dencie Desrosiers, Stephanie S. Lee, Alexander G. Shtukenberg,* Bart Kahr*[a]

[*] Department of Chemistry and Molecular Design Institute
New York University
24 Waverly Place, New York, NY 10003, USA
E-mail: bk66@nyu.edu

Table of Contents

S1 – Refinement details

S2 – Triphenylmethyl cation geometries

S3 – Disorder in structure **2** polyhalogen network

S4 – Alternative structure **3** refinement details

S5-7 – Raman spectroscopy data and experimental details

S8 – Powder X-ray Diffraction details

S1) Deposited Structure Refinement Details

Structure	**1**	**2**	**2**	**3**
Formula	$C_{19}H_{15}Br_2I_3$	$C_{17.72}H_{14.72}Br_{0.14}I_{5.45}$	$C_{17.58}H_{14.58}Br_{0.23}I_{5.41}$	$C_{38}H_{30}Br_3I_9$
Space Group	*Pnna*	$P\bar{3}c1$	$P\bar{3}c1$	*C*2/*c*
a, Å	17.9244(7)	16.4645(4)	16.4316(5)	25.553(2)
b, Å	12.6300(5)	16.4645(4)	16.4316(5)	25.635(2)
c, Å	9.4066(3)	19.1960(7)	19.1660(9)	29.682(2)
β, °	90	90	90	95.687(3)
V, Å^3	2129.5(1)	4506.5(3)	4481.5(3)	19347(2)
μ, mm^{-1}	8.155	7.772	7.915	8.272
D_x, g/cm^3	2.445	2.743	2.759	2.566
2ϑ Range, °	2.27 – 28.30	2.12 – 28.36	1.43 – 28.56	1.59 – 28.35
T, K	100(2)	100(2)	99(2)	100(2)
Total reflns.	2655	3757	3754	24037
Obs. reflns. [$I > 2\sigma_I$]	2346	3555	3495	24046

R_1 [$I > 2\sigma_I$], %	2.12	3.65	4.33	8.73
wR_2 [all data], %	4.92	8.66	11.38	22.39
GoF	1.131	1.277	1.160	1.158
Number of Parameters	111	194	179	902
Number of Restraints	0	2	12	36
Deposition Number	2431723	2431721	2431722	2505334

Table S1. All structure refinement and deposition details.

S2) Triphenylmethyl Cation Geometries

TPM Name	Dihedral Angles (º)	C-C_{methyl}-C Angles (º)
1	45.7(2)	122.6(2)
	27.4(2)	118.7(2)
	27.4(2)	118.7(2)
2	38.0(2)	121.4(2)
	28.0(2)	119.3(2)
	28.0(2)	119.3(2)
3_{C7}	38.1(5)	121.7(5)
	31.0(5)	120.3(5)
	30.3(5)	118.0(5)
3_{C26}	37.4(5)	121.0(5)
	32.8(5)	120.0(5)
	28.7(5)	119.1(5)
3_{C45}	38.0(5)	120.7(5)
	31.6(5)	120.3(5)
	31.1(5)	119.0(5)
3_{C64}	39.0(5)	120.8(5)
	30.9(5)	120.7(5)
	29.5(5)	118.5(5)

Table S2. All unique TPM cation geometries, with angles listed in descending order. Dihedral angles measured between the average plane bisecting the central carbon and surrounding 3 carbons, and the average plane bisecting all 6 carbons on each phenyl ring, calculated in Mercury software. Structure **3** cations named by central carbon in .cif file.

S3) Disorder in Structure **2** Iodine Network

Structure **2** features a complex disordered iodine network. Visualizing this disorder gives a better understanding of how and where iodine is distributed within the 3D network.

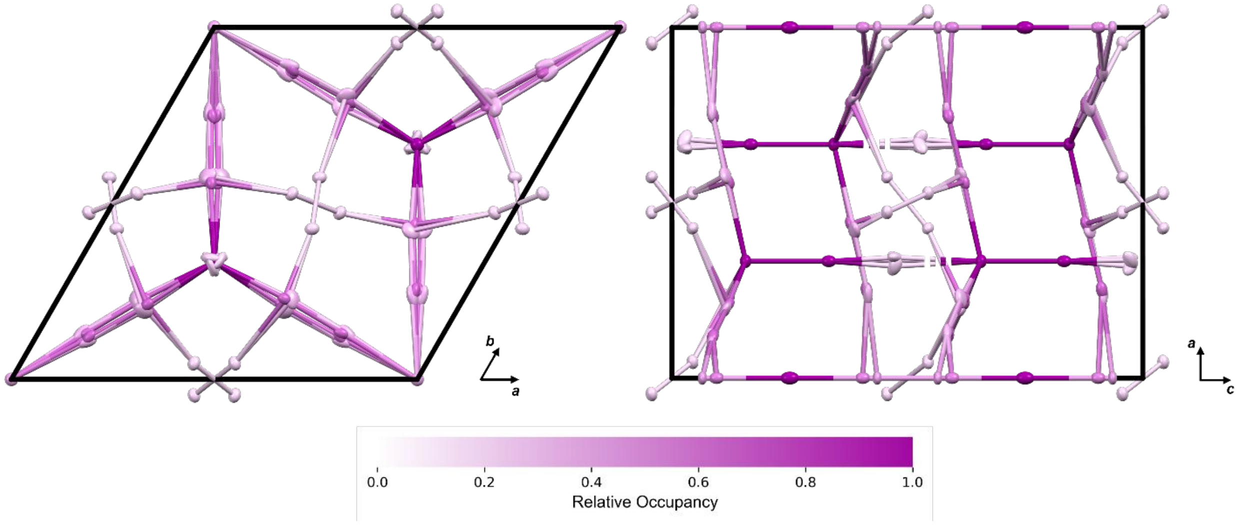

Figure S3. Structure **2** iodine network viewed along *c* (left) and *b* (right) axes. Iodine atoms in ellipsoidal style colored in accordance with their occupancies.

S4) Alternative Structure **3** Solution

To reckon with the residual electron density around the TPM phenyl rings, an alternative refinement (summarized below) includes partial occupancy (0.089(2)) of I_2 molecules with I-I bond lengths (2.70(2) - 2.73(2) Å) overlay one of the triphenylmethyl rings in each independent triphenylmethyl cation. The superposition is unphysical and is a likely consequence of unresolved twinning.

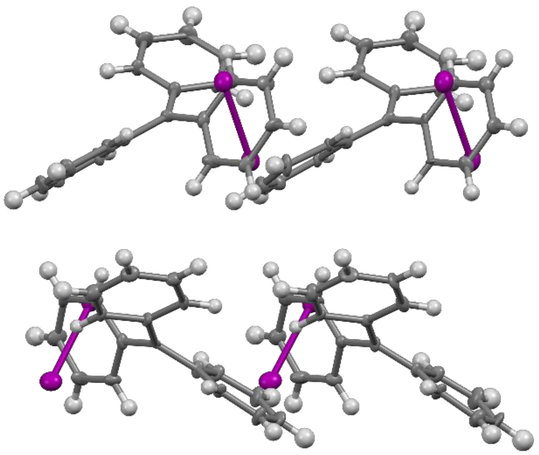

Figure S4. For alternate structure **3** refinement, viewed along *a*, all atoms stylized as ellipsoids. Iodine diatoms bisect TPM phenyl rings in an unphysical manner.

Structure	**3**	**3** (Alternate Refinement)
Formula	$C_{38}H_{30}Br_3I_9$	$C_{38}H_{30}Br_3I_9$
Space Group	*C*2/*c*	*C*2/*c*
a, Å	25.553(2)	25.553(2)
b, Å	25.635(2)	25.635(2)
c, Å	29.682(2)	29.682(2)
β, º	95.687(3)	95.687(3)
V, $Å^3$	19347(2)	19347(2)
μ, mm^{-1}	8.272	8.495
2ϑ Range, º	1.59 – 28.35	1.59 – 28.35
D_x, g/cm^3	2.566	2.627
T, K	100(2)	100(2)
Total reflns.	24037	24043
Obs. reflns. [$I > 2\sigma_I$]	24046	21663
R_1 [$I > 2\sigma_I$], %	8.73	7.01
wR_2 [all data], %	22.39	17.37
GoF	1.158	1.216
Number of Parameters	902	975
Number of Restraints	36	0
Deposition Number	2505334	Not Deposited

Table S4. All details of alternative structure **3** refinement described.

S5-7) Raman Spectroscopy

I) Procedure

A Thermo Fischer Scientific DXR2 Raman Microscope equipped with DXR 785 nm LASER, filter, and 830 lines/mm grating was utilized. An MPlan 10x/0.25 objective was selected, laser power was kept at 5 mW to minimize damage to crystals and loss of iodine. 30 spectra were averaged, and three repeat runs were performed on different crystals of each morphology. White light and fluorescence correction (polynomial order 2) was utilized, alongside medium cosmic ray threshold and smart background.

II) Spectra

Consistent with literature,[1] halogenic Raman activity takes place below 200 cm^{-1}. TPM cation Raman activity presents above this wavenumber in all samples.

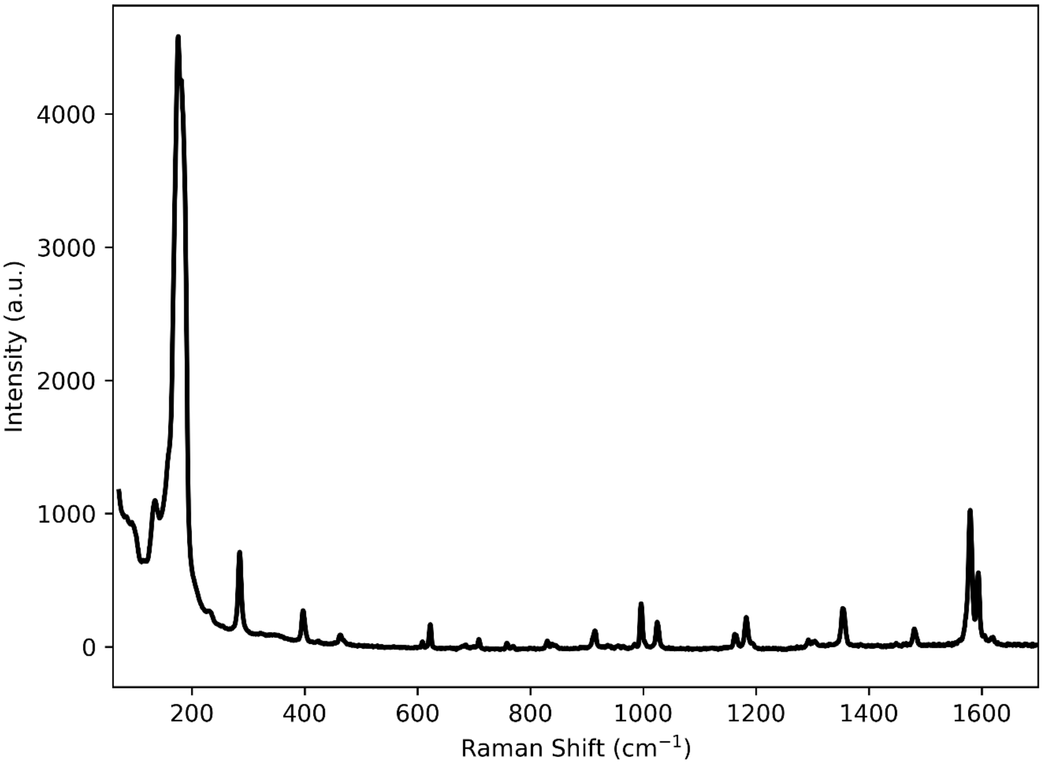

Figure S5. Raman spectrum of needle morphology crystal (Structure **1**).

Raman Shift (cm^{-1})	Intensity (a.u.)
134.9	1098.4
175.9	4581.5
181.7	4252.2
284.9	710.8
397.2	272.5
463.1	84.5
608.8	38.5
622.8	167.2
708.6	56.0
758.3	28.7
769.4	5.1
830.1	44.8
914.5	120.3
996.4	324.5
1024.9	185.4
1162.3	94.8
1164.7	88.1

1182.5	220.6
1292.9	53.6
1353.7	288.9
1480.5	135.2
1579.8	1025.4
1594.3	556.6

Table S5. Indexed Raman peaks of needle morphology crystal (Structure **1**).

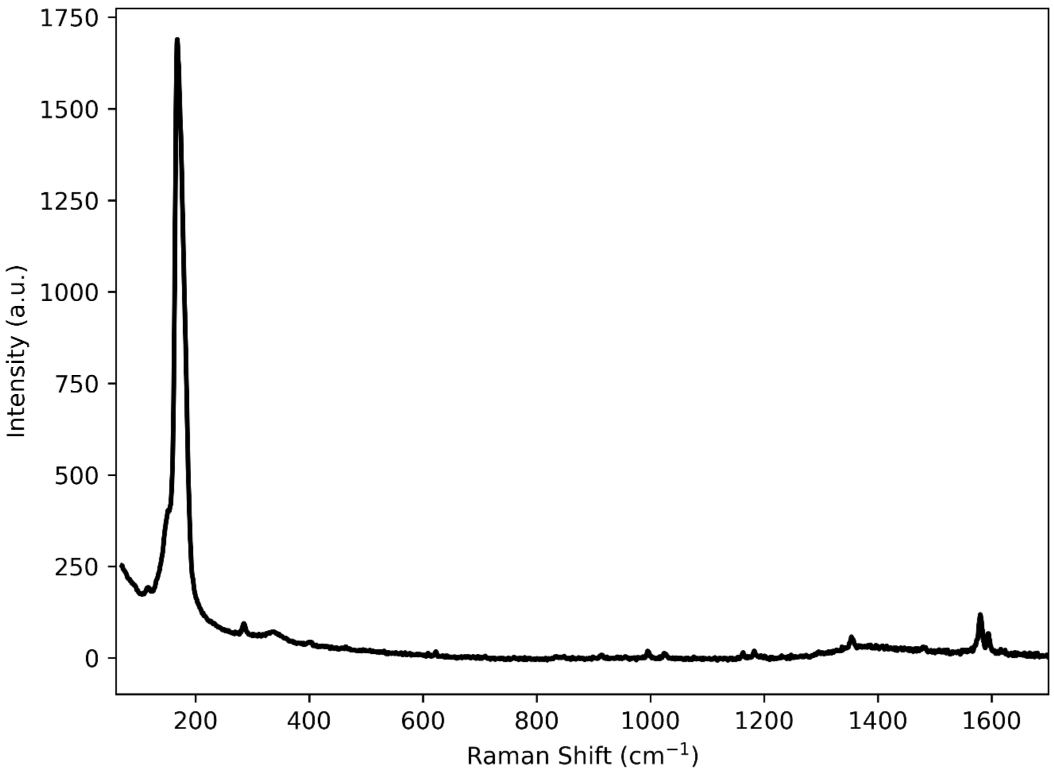

Figure S6. Raman spectrum of hexagonal prism morphology crystal (Structure **2**).

Raman Shift (cm^{-1})	Intensity (a.u.)
167.9	1689.3
284.8	94.2
622.8	17.3
995.0	18.8
1024.4	12.6
1163.2	14.8
1182.5	19.0
1353.7	57.7
1580.3	118.5
1594.3	66.4

Table S6. Indexed Raman peaks of hexagonal prism morphology crystal (Structure **2**).

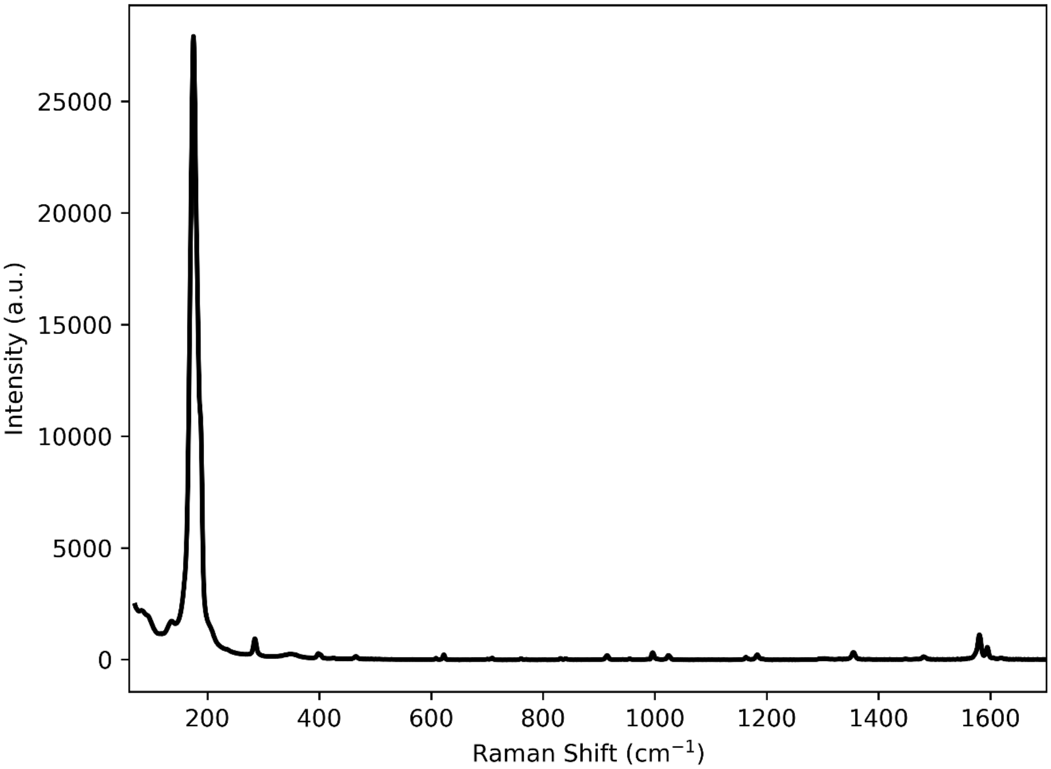

Figure S7. Raman spectrum of plate morphology crystal (Structure **3**).

Raman Shift (cm^{-1})	Intensity (a.u.)
135.9	1721.8
174.9	27908.9
284.8	931.0
398.1	268.6
465.7	140.0
608.8	58.0
622.8	213.6
709.1	65.0
760.9	38.8
770.3	13.2
831.1	50.8
840.2	30.3
914.5	179.7
956.0	26.4
996.0	305.5
1024.4	118.4
1162.8	102.6
1183.0	212.5
1355.1	316.3
1480.5	124.5
1580.3	1100.0
1594.3	550.0

Table S7. Indexed Raman peaks of plate morphology crystal (Structure **3**).

S8-9) Powder X-ray Diffraction

I) Procedure

Crude crystals were taken in aggregate directly from benzene solution, powdered with mortar and pestle, and flattened on a glass sample holder. The powder diffraction pattern was collected in Bragg-Brentano geometry with a Rigaku SmartLab rotating anode 9 kW system using Cu$K\alpha$ radiation. Four successive datasets were collected with 2ϑ = 5 to 35° at a rate of 3 °/min and a step size 0.01° and then combined. Pawley fitting and Rietveld refinement were performed using TOPAS software.[2]

For Pawley fitting, the match is excellent (R_{wp} = 1.46%), and the difference curve is flat. All observed diffraction maxima are accounted for by one of three phases, except for one small peak around 8.3, which could represent a different crystalline phase, but it is not sufficient to substantiate this proposition. Due to the large number of possible reflections from the three unique phases, the fit of the unit cell dimensions (especially for the plate, phase **3**) is not well defined.

We also performed Rietveld refinement (R_{wp} = 13.926%). The intensities do not match well, likely due to texture or differences in crystal structure. Phase concentrations are 55.1%, 25.6%, and 19.3% for phases **1**, **2**, and **3**, respectively. This composition is consistent with our elemental analysis.

II) Results

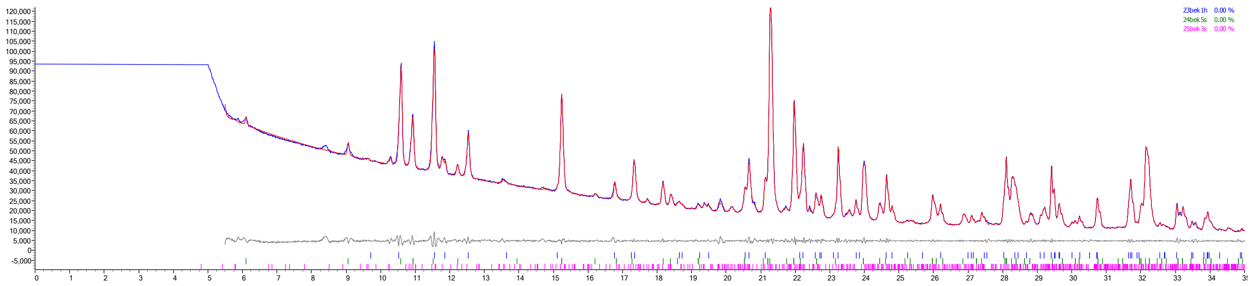

Figure S8. Pawley fit of structures **1** (blue tick marks) **2** (green tick marks) and **3** (magenta tick marks) on powder data collected on crude mixture of Gomberg's crystals. R_{wp} = 1.46%. Experimental data in blue, Pawley fit in red, and difference curve plotted below in gray.

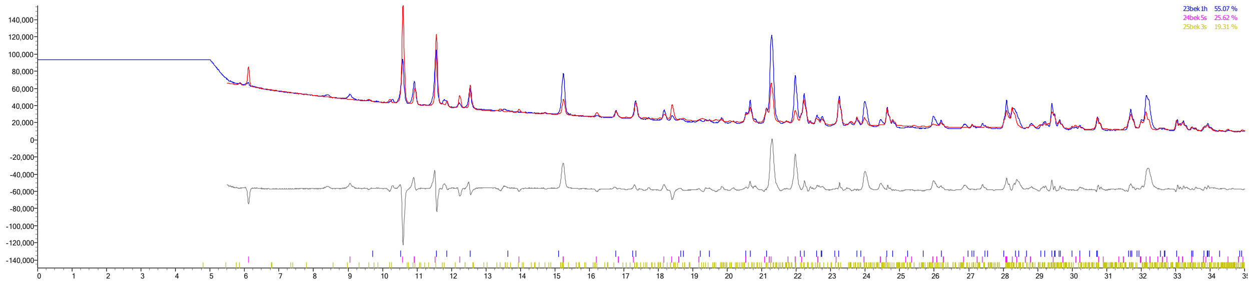

Figure S9. Rietveld refinement on same powder data collected on crude mixture of Gomberg's crystals. R_{wp} = 13.93%. Experimental data in blue, Pawley fit in red, and difference curve plotted below in gray. Phase concentrations reported of 55.1%, 25.6%, and 19.3% for structures **1** (blue tick marks), **2** (magenta tick marks) and **3** (orange tick marks), respectively.

All data is available upon request.

References

[1] P. Deplano, F. A. Devillanova, J. R. Ferraro, M. L. Mercuri, V. Lippolis, and E. F. Trogu, *Applied Spectroscopy*, **1994**, *48*, 1236–1241.

[2] Bruker AXS. TOPAS V4: General profile and structure analysis software for powder diffraction data. User's Manual, Bruker AXS, Karlsruhe, Germany, 2008.
